# Longitudinal associations between changes in employment status and depressive symptoms during the early COVID-19 pandemic: evidence from the Canadian Longitudinal Study on Aging (CLSA)

**DOI:** 10.24095/hpcdp.46.4.03

**Published:** 2026-04

**Authors:** Brianna Frangione, Ying Jiang, Margaret de Groh, Esme Fuller-Thomson, Ian Colman, Paul J. Villeneuve

**Affiliations:** 1 Department of Neuroscience, Carleton University, Ottawa, Ontario, Canada; 2 Applied Research Division, Centre for Surveillance and Applied Research, Public Health Agency of Canada, Ottawa, Ontario, Canada; 3 School of Epidemiology and Public Health, University of Ottawa, Ottawa, Ontario, Canada; 4 Factor-Inwentash Faculty of Social Work, University of Toronto, Toronto, Ontario, Canada; 5 Institute for Life Course and Aging, University of Toronto, Toronto, Ontario, Canada; 6 CHAIM Research Centre, Carleton University, Ottawa, Ontario, Canada

**Keywords:** cohort study, employment status, depression, older adults, COVID-19

## Abstract

**Introduction:**

The COVID-19 pandemic caused unprecedented and inequitably distributed adverse health impacts, which varied across socioeconomic circumstances. We investigated differences in incident depression among individuals aged 50 years and older according to various employment factors during the early stages of the pandemic.

**Methods:**

We included 16 719 Canadian Longitudinal Study on Aging participants who provided data at Follow-up one (2015–2018) (FUP1) and twice during the pandemic (Spring and Autumn 2020). The Center for Epidemiologic Studies Depression Scale (CESD-10) was used to classify individuals with depression (CESD-10 score≥10). Logistic regression, adjusted for possible confounders, estimated the odds of incident depression in Autumn 2020.

**Results:**

We found depression scores worsened from pre-pandemic (FUP1) to Autumn 2020; this pattern was evident across different employment features. Individuals who were newly unemployed in Spring 2020 had over double the odds of depression in Autumn 2020 (odds ratio [OR]=2.22; 95% confidence interval [CI]: 1.51–3.28) compared to those who remained retired. Higher odds of depression were also observed among those with employment disruptions in Spring 2020 relative to those who did not (OR=1.65; 95% CI: 1.28–2.12), and individuals primarily working in non-home-based settings in Autumn 2020 had 21% lower odds of depression (OR=0.79; 95% CI: 0.63–0.98) than those who worked remotely.

**Conclusion:**

Our findings suggest that employment status was an important predictor of depression among Canadians during the early phases of the pandemic.

**Highlights**

- Employment disruptions and unemployment during the pandemic significantly increased the odds of developing depression, highlighting the need for targeted mental health support for affected groups.
- Newly unemployed individuals had 122% higher odds of developing depression than retired individuals.
- Remote workers experienced greater increases in depression compared to those working in non-home-based settings.
- During the early stages of the pandemic, women experienced larger increases in depression scores compared to men.
- Individuals with chronic health conditions, younger age, and lower income had higher depression scores.

## Introduction

Employment status is an important and often overlooked social determinant of health that impacts health in numerous ways.^1^ For many, employment is a critical component of social identity and provides the structure and opportunities for social interactions.^2^,^3^ It also follows that unemployment may affect mental well-being adversely,^2^ with unemployed individuals reporting lower well-being than their employed counterparts.^4^

During the COVID-19 pandemic, public health measures such as strict lockdowns, physical distancing, and isolation led to a substantial rise in mental illness.^5^ Additionally, business closures and reduced working hours increased unemployment in Canada and other countries,^6^ contributing to personal stress and exacerbating mental health issues.^7^ In Canada, socioeconomic factors, including lower income and unstable working hours, were major drivers of anxiety during the pandemic.^8^ Evidence on the mental health impacts of different work arrangements has been mixed. In a Canadian cross-sectional study, Bodner et al.^9^ found that those who worked exclusively from home or in person reported poorer self-rated mental health than those who worked in a hybrid arrangement; however, only 13% of their cohort was 50 years of age or older. Elsewhere, Beland et al.^6^ noted that deteriorations in mental health among Canadian workers were less severe for essential workers, men, and those who could work remotely. Additionally, several studies have reported that the mental health effects of COVID-19 were greater for women compared to men.^10^-^13^ However, a Korean study evaluated the prevalence of depression before and during the pandemic and reported an increase in depression prevalence in men compared to women, who showed no differences in depressive symptoms between time points.^14^

Outside of Canada, numerous studies have evaluated the impacts of the pandemic on mental health by employment status.^15^-^23^ Several studies reported higher rates of burnout among essential workers,^15^,^16^ including medical professionals,^17^-^19^ and there may be important gender differences in these effects,^20^ with females experiencing higher levels than males.^21^ Most of these studies have targeted specific occupations and relied on cross-sectional study designs, which are less robust than longitudinal designs. In contrast to Bodner et al.,^9^ a Finnish cohort study^22^ found that individuals who worked from home had improved perceptions of psychosocial work environment, compared to those in the workplace. Additionally, Wester et al.^23^ found decreased sadness and depression among employed and retired participants in a cohort of approximately 36000 Europeans when compared to pre-pandemic levels.^23^

In Canada, few studies have used longitudinal data to explore the effects of employment status on depression during the early stages of the pandemic. To address this gap, we analyzed data from the Canadian Longitudinal Study on Aging (CLSA), a comprehensive health survey conducted before and twice during the early phase of the pandemic. This survey offers a unique opportunity to assess the effects of employment status on depression in individuals aged 50 and older. Our primary objective was to examine the occurrence of depressive symptoms during this period based on employment circumstances. Additionally, we explored whether these associations varied between men and women. 

## Methods

***Study population***

The CLSA comprises a sample of 51 338 individuals recruited from Canadian provinces between 2011 and 2015. At the time of enrolment, these men and women were 45 and 85 years old, could complete the questionnaires in English or French, and were physically and cognitively able to provide consent and participate independently. The CLSA is comprised of two cohorts: the Comprehensive and the Tracking. Those in the Comprehensive cohort participated via in-home interviews and visits to one of the 11 data collection sites for physical and cognitive examinations and optional blood and urine tests. Those in the Tracking cohort were administered the questionnaires via computer-assisted telephone interviews. The Comprehensive and Tracking cohorts were combined in this study. Detailed descriptions of the CLSA design are published elsewhere.^24^

In this study, we used the CLSA data from four waves of questionnaires: the Baseline (2011 to 2015), Follow-up one (2015 to 2018) (hereafter FUP1), COVID-19 Baseline (hereafter Spring 2020), and COVID-19 Exit (hereafter Autumn 2020). The Baseline and FUP1 questionnaires collected data on sociodemographic factors, physical and mental health, and behaviour. The COVID-19 questionnaires were launched to investigate the health effects of the pandemic. All the CLSA participants were invited to partake in the Spring 2020 COVID-19 study, of which 42700 were alive and did not require a proxy to complete the questionnaire. There were 28 559 (67.2%) individuals who agreed to participate. Additionally, the response rate for the Autumn 2020 questionnaire was 84.4%. 

***Study sample***

**Figure 1:**
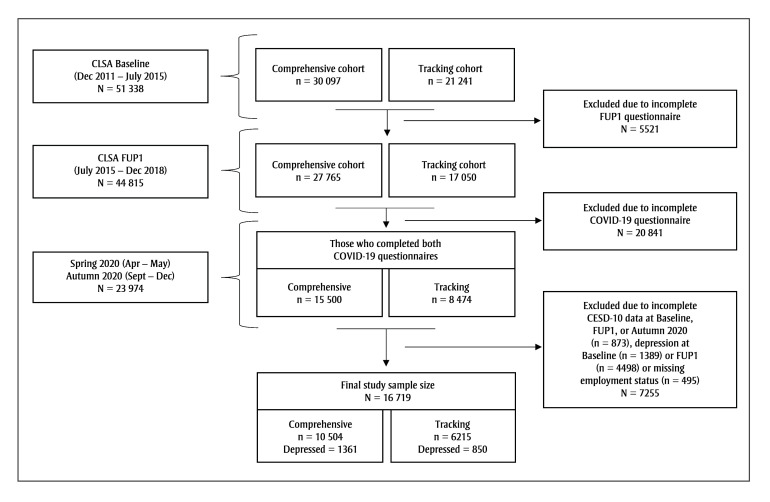
Flow diagram outlining the number of participants of the Canadian Longitudinal Study on Aging that comprised the analysis file

**Abbreviations: **CLSA, Canadian Longitudinal Study on Aging; FUP1, Follow-up one. 

**Note: **Individuals were classified as having depression when CESD-10 score was greater than or equal to 10. 

The diagram outlining the number of participants that comprised the analysis file is shown in Figure 1. The initial sample included 23  974 participants who completed the Spring 2020 and Autumn 2020 questionnaires. The Center for Epidemiological Studies Short Depression Scale (CESD-10),^25^ a validated self-report measure, was included in each survey wave. There were 7255 participants excluded due to incomplete CESD-10 scores (n=873) at Baseline, FUP1, or Autumn 2020 surveys, or had depression scores greater than or equal to 10 at Baseline (n=1389), or FUP1 (n=4498). The final number of participants was 16  719.

***Measurement of employment status***

The CLSA survey instrument included several items that allowed respondents to describe their employment status. The time points of the variable assessments are provided in Table 1. In this study, the term “employed” refers exclusively to paid employment, as defined by self-reported current work participation and schedule. Unpaid work, such as caregiving or volunteer activities, was not captured within our employment status variables. Employment status definitions combined self-reported retirement status, current work participation, work schedule, and workplace circumstances to create categorical employment variables across survey waves. 

**Table 1:** Time points of variable assessments, Canadian Longitudinal Study on Aging, 2020, Canada

Characteristic	Baseline	FUP1	Spring 2020	Autumn 2020
Sex	Yes	No	No	No
Age (years)	No	No	Yes	No
Marital status	No	Yes	No	No
Household income	No	Yes	No	No
Highest attained education	No	Yes	No	No
Mortgage status	No	Yes	No	No
Smoking status in Spring 2020	No	No	Yes	No
Alcohol consumption since 1 March 2020	No	No	No	Yes
Chronic condition status	No	Yes	No	No
Employment status at FUP1	No	Yes	No	No
Employment status in Autumn 2020	No	No	No	Yes
Primary work location	No	No	No	Yes
Essential worker status	No	No	Yes	No
Work disrupted in the past 30 days	No	No	Yes	No
Depression survey (CESD-10)	Yes	Yes	No	Yes

**Abbreviations: **CESD-10, Center for Epidemiological Studies Short Depression Scale; FUP1, Follow-up one. 

Subjective retirement status was considered at FUP1, where participants were classified as fully retired, not retired, partly retired, or never having worked. Those who were not retired or partly retired were further distinguished by current work participation. Individuals who were currently working were categorized as working full-time or part-time based on their work schedule. Respondents who indicated they were not currently working were assigned unemployed if they reported unemployment as the reason or “other reason” otherwise, which included reasons such as disability and caring for family. 

In Spring 2020, employment status was determined by combining FUP1 status with questions on employment location. Participants working in non-home-based settings were categorized as working in the workplace, switched to remote work, or had their workplace closed, depending on their responses to workplace distancing implementation items. Those in home-based settings were classified as fully retired, working remotely, unemployed, not working otherwise, or never worked, conditional on their FUP1 status. The Autumn 2020 classification followed a similar process. Retirement was carried forward where applicable, while active workers were defined jointly by work schedule and location. Categories included full-time or part-time work either remotely, in the workplace, or “other.” The latter group may represent individuals with hybrid work arrangements, although this cannot be definitively determined from the available data. Respondents who reported being unemployed or retired were classified accordingly. 

To capture changes in employment during the pandemic, we derived employment transition variables indicating changes in survey waves (Spring 2020 to Autumn 2020 and FUP1 to Autumn 2020). Transitions were constructed only for participants with non-missing employment status at each survey wave. Transitions between survey waves were classified in stable trajectories, directional shifts, pandemic-related disruptions, and changes in intensity. Stable trajectories captured individuals who remained in the same category across waves, including those who remained retired, remained in the workplace, or remained working remotely. Directional shifts reflected entry into or exit from the labour force, which included individuals who were newly retired (transitioned from employment to retirement), newly employed (entered employment after being unemployed or not working), or newly unemployed (moved from employment to unemployment). Pandemic-related disruptions included temporary workplace closures and changes in work modality. Workplace closure included individuals who were employed at one survey wave but were not working due to workplace shutdowns at the next survey wave. Those who returned to the workplace after closure included individuals who resumed on-site work following the closure of their workplace. Additional categories included individuals who switched to remote work and those who returned to the workplace after working remotely. Changes in work intensity identified shifts in working hours irrespective of location: increased hours (part-time to full-time) and reduced hours (full-time to part-time). Finally, the “other” category represents categories that did not fit the prespecified definitions or those with small sample sizes, such as individuals who remained unemployed or returned from retirement. 

In addition to the transition variables, essential worker status was assessed in Spring 2020 with the question, “Are you considered an essential worker?”. Whether one’s employment was disrupted in the past 30 days was assessed in Spring 2020. Furthermore, participants were asked in Autumn 2020 to indicate whether their primary employment location was non-home based. 

***Measurement of depression***

The CLSA surveys include the CESD-10^25^ instrument, which is a validated self-report screening tool for depressive symptoms. The CESD-10 is comprised of 10 questions that allow for the identification of feelings related to depression, loneliness, and happiness. Responses were summed to give a final score between zero and 30. Previous research indicates that a scoregreater than or equal to10 has sufficient sensitivity and specificity to identify depression.^25^,^26^ Therefore, CESD-10 scores were classified into a dichotomous outcome, and participants with a cumulative score of 10 or higher were classified as having depression. It is important to note that the CESD-10 does not provide a definitive clinical diagnosis of depression; rather, it identifies individuals with elevated depressive symptomology. In Canada, only qualified health professionals (e.g. physicians, psychiatrists, or psychologists) can diagnose depression. Lifetime prevalence of depression was measured at FUP1 using the question, “Has a doctor ever told you that you suffer from clinical depression?”. This question was only asked of the Comprehensive cohort participants. 

***Covariates***

Demographic factors included sex at Baseline, age in Spring 2020, and marital status at FUP1. Socioeconomic factors included household income, highest education achieved, and mortgage status, assessed at FUP1. Smoking status and alcohol consumption were assessed in Spring 2020 and Autumn 2020, respectively. Chronic condition status was assessed at FUP1. Although physical activity data were collected in the CLSA, these measures were only available at FUP1 and not during the Spring 2020 and Autumn 2020 surveys. Because FUP1 data were collected several years before the pandemic and were based on the Physical Activity Scale for the Elderly, which captures typical weekly activity, we did not consider these data a reliable covariate for pandemic analyses. Moreover, evidence shows that physical activity patterns changed substantially during the pandemic,^27^ making pre-pandemic measures unlikely to accurately reflect activity during the study period. 

***Statistical methods***

The mean CESD-10 scores were reported across all covariates and employment indicators at Baseline, FUP1, and Autumn 2020. Additionally, the mean difference was reported using the individual paired data, representing the mean change in CESD-10 scores from Baseline to FUP1, and FUP1 to Autumn 2020. A likelihood ratio test was performed to test for interaction with sex, which was not statistically significant. However, we repeated the analyses stratified by sex to present findings separately for men and women. 

Unconditional logistic regression was used to calculate odds ratios (ORs) with 95% confidence intervals (CIs) to estimate the odds of incident depression (CESD-10 score≥10) in Autumn 2020 for each employment status indicator. A complete-case approach was used for the logistic regression models, and only a single employment indicator was included in the model at any one time. Only individuals without depression (CESD-10 score<10) at Baseline or FUP1 were retained for analyses of incident depression in Autumn 2020. The final model was adjusted for age, sex, marital status, household income, highest education, mortgage status, chronic conditions, smoking habits, and frequency of alcohol consumption. Additionally, those who had depression at Baseline or FUP1 but not in Autumn 2020 were examined to assess if employment status was associated with remission. The remission analysis was adjusted for the same covariates listed above. Statistical significance was determined at a *p*≤.05. Data were analyzed using SAS software 9.4.^28^

The STROBE Cohort Reporting Guidelines were used when writing this manuscript.^29^

***Ethics approval and consent to participate***

The secondary analysis for this study was approved by the University of Toronto Research Ethics Board (Protocol #41167).

## Results

In our study sample of 16 719 CLSA participants aged 50 and older, there were approximately equal proportions of men (50.4%) and women (49.6%) (Table 2). Women, on average, had higher CESD-10 scores at all time points, and larger average increases in depression scores from FUP1 to Autumn 2020, compared to men; this pattern was consistent across nearly all descriptive characteristics (data not shown). There was a consistent inverse relationship between age and average changes in CESD-10 scores, such that younger individuals experienced larger increases in depression scores between FUP1 and Autumn 2020. Additionally, higher average changes in depression scores were observed for those who were single, those with chronic health conditions, and those who smoked and consumed alcohol regularly. Higher-income individuals had larger average increases in depression scores from FUP1 to Autumn 2020; however, there was an inverse relationship between income and CESD-10 scores across all individual time points. Importantly, across nearly all characteristics, there was a worsening of depression scores from FUP1 to Autumn 2020, whereas there was an improvement of depression scores from Baseline to FUP1. 

**Table 2:** Means and standard errors (SE) of CESD-10 scores at Baseline, Follow-up one (FUP1), Autumn 2020, and the mean change in scores from Baseline to FUP1, and FUP1 to Autumn 2020, according to different covariates in individuals without depression at Baseline or FUP1 (sample size = 16 719; incident cases in Autumn 2020 = 2211)

Characteristic		Sample size	Mean (SE) Baseline CESD-10 score	Mean (SE) FUP1 CESD-10 score	Mean (SE) Autumn 2020 CESD-10 score	Mean (SE) change in CESD-10 scores from Baseline to FUP1	Mean (SE) change in CESD-10 scores from FUP1 to Autumn 2020
Total		16 719	3.30 (0.02)	3.25 (0.02)	4.86 (0.03)	−0.06 (0.02)	1.61 (0.03)
Sex	Male	8 434	3.19 (0.03)	3.12 (0.03)	4.47 (0.04)	−0.08 (0.03)	1.36 (0.04)
	Female	8 285	3.42 (0.03)	3.38 (0.03)	5.25 (0.05)	−0.04 (0.03)	1.87 (0.05)
Age (years)	50–54	744	3.52 (0.09)	3.22 (0.09)	5.76 (0.18)	−0.30 (0.10)	2.55 (0.17)
	55–59	2 257	3.39 (0.05)	3.21 (0.05)	5.15 (0.09)	−0.18 (0.06)	1.94 (0.09)
	60–64	2 666	3.42 (0.05)	3.15 (0.05)	4.74 (0.08)	−0.27 (0.05)	1.59 (0.08)
	65–69	3 194	3.26 (0.04)	3.13 (0.04)	4.71 (0.07)	−0.13 (0.05)	1.58 (0.07)
	70–74	2 962	3.16 (0.04)	3.15 (0.05)	4.60 (0.07)	−0.01 (0.05)	1.46 (0.07)
	≥ 75	4 896	3.28 (0.04)	3.46 (0.04)	4.89 (0.06)	0.18 (0.04)	1.44 (0.06)
Marital status	Single/never married	1 195	3.70 (0.07)	3.55 (0.07)	5.36 (0.13)	−0.15 (0.08)	1.81 (0.13)
	Married/common-law	12 414	3.19 (0.02)	3.13 (0.02)	4.72 (0.04)	−0.06 (0.02)	1.59 (0.04)
	Widowed/separated/divorced	3 102	3.61 (0.05)	3.59 (0.05)	5.20 (0.08)	−0.02 (0.05)	1.60 (0.08)
	Missing	8	3.07 (0.98)	2.13 (0.44)	5.88 (1.46)	−0.94 (0.80)	3.75 (1.39)
Highest attained education	No post-secondary	1 166	3.46 (0.07)	3.47 (0.07)	5.02 (0.12)	0.02 (0.08)	1.55 (0.12)
	Any post-secondary	9 464	3.34 (0.03)	3.25 (0.03)	4.94 (0.04)	−0.09 (0.03)	1.70 (0.04)
	Above post-secondary	3 814	3.04 (0.04)	3.00 (0.04)	4.75 (0.07)	−0.03 (0.04)	1.75 (0.06)
	Missing	2 275	3.52 (0.05)	3.53 (0.05)	4.58 (0.09)	0.01 (0.06)	1.05 (0.08)
Household income	≤ $20 000	402	3.91 (0.12)	3.98 (0.13)	4.84 (0.22)	0.07 (0.15)	0.87 (0.21)
	$20 000–$50 000	2 995	3.63 (0.05)	3.61 (0.05)	5.01 (0.08)	−0.03 (0.05)	1.40 (0.08)
	$50 000–$100 000	6 091	3.28 (0.03)	3.26 (0.03)	4.89 (0.05)	−0.01 (0.03)	1.62 (0.05)
	$100 000–$150 000	3 449	3.18 (0.04)	3.04 (0.04)	4.80 (0.07)	−0.14 (0.04)	1.76 (0.06)
	≥ $150 000	2 941	3.06 (0.04)	2.89 (0.04)	4.66 (0.08)	−0.16 (0.05)	1.77 (0.07)
	Missing	841	3.39 (0.08)	3.53 (0.09)	4.98 (0.15)	0.14 (0.10)	1.46 (0.15)
Dwelling location	Rural	1 700	3.17 (0.06)	3.11 (0.06)	4.47 (0.10)	−0.06 (0.07)	1.36 (0.09)
	Urban	15 011	3.32 (0.02)	3.26 (0.02)	4.90 (0.03)	−0.06 (0.02)	1.64 (0.03)
	Missing	8	4.38 (1.15)	4.63 (1.02)	3.93 (0.75)	0.25 (1.00)	−0.69 (1.12)
Comorbidity status	No chronic condition	701	2.72 (0.09)	2.51 (0.09)	3.86 (0.14)	−0.22 (0.09)	1.35 (0.14)
	At least one chronic condition	15 810	3.33 (0.02)	3.29 (0.02)	4.91 (0.03)	−0.05 (0.02)	1.62 (0.03)
	Missing	208	2.93 (0.17)	2.71 (0.15)	4.28 (0.26)	−0.22 (0.16)	1.58 (0.25)
Smoking status in Spring 2020	Never	15 755	3.29 (0.02)	3.23 (0.02)	4.84 (0.03)	−0.06 (0.02)	1.61 (0.03)
	Occasional	190	3.64 (0.18)	3.57 (0.18)	5.00 (0.33)	−0.08 (0.19)	1.44 (0.32)
	Daily	675	3.46 (0.10)	3.36 (0.10)	5.09 (0.17)	−0.10 (0.11)	1.74 (0.17)
	Missing	99	3.92 (0.27)	3.89 (0.28)	5.24 (0.41)	−0.03 (0.30)	1.35 (0.44)
Alcohol consumption since 1 March 2020	Never	2 772	3.37 (0.05)	3.38 (0.05)	4.57 (0.08)	0.01 (0.05)	1.19 (0.08)
	1–3 times per month	4 563	3.43 (0.04)	3.37 (0.04)	4.91 (0.06)	−0.06 (0.04)	1.54 (0.06)
	1–5 times per week	6 636	3.21 (0.03)	3.13 (0.03)	4.88 (0.05)	−0.07 (0.03)	1.75 (0.05)
	Almost daily	2 727	3.26 (0.05)	3.18 (0.05)	5.00 (0.08)	−0.08 (0.05)	1.82 (0.08)
	Missing	21	3.86 (0.45)	3.71 (0.57)	5.12 (0.99)	−0.14 (0.49)	1.40 (0.67)

**Abbreviation:** CESD-10, Center for Epidemiological Studies Short Depression Scale. 

**Note: **Higher CESD-10 scores indicate more severe depressive symptoms. Individuals were classified as having depression when CESD-10 score was greater than or equal to 10. 

Table 3 presents data related to mean changes in depression scores from FUP1 to Autumn 2020 across various employment characteristics. While increases in depressive symptoms were observed across all employment circumstances, the largest increase was observed for those who became newly unemployed in Spring 2020. Similarly, larger changes in depression scores were observed for those whose workplace closed in Spring 2020 or Autumn 2020, and for those who switched to remote work. Additionally, there were smaller increases in depression scores among those who worked primarily in person, non-essential workers, and those whose work was not disrupted in Spring 2020. Following stratification by sex, women, on average, had larger increases in CESD-10 scores from FUP1 to Autumn 2020, compared to men, across nearly all employment measures (data not shown). 

**Table 3:** Means and standard errors (SE) of CESD-10 scores at Baseline, Follow-up one (FUP1), Autumn 2020, and the mean change in scores from Baseline to FUP1 and FUP1 to Autumn 2020, according to employment situation in individuals without depression at Baseline or FUP1 (sample size = 16 719; incident cases in Autumn 2020 = 2211)

Employment situation		Sample size	Mean (SE) Baseline CESD-10 score	Mean (SE) FUP1 CESD-10 score	Mean (SE) Autumn 2020 CESD-10 score	Mean (SE) change in CESD-10 scores from Baseline to FUP1	Mean (SE) change in CESD-10 scores from FUP1 to Autumn 2020
Change from Spring 2020 to Autumn 2020	Remained retired	9 184	3.29 (0.03)	3.31 (0.03)	4.82 (0.04)	0.02 (0.03)	1.51 (0.04)
	Newly retired	264	3.05 (0.15)	3.28 (0.15)	4.40 (0.25)	0.23 (0.15)	1.12 (0.25)
	Newly employed	293	3.63 (0.14)	3.72 (0.15)	5.26 (0.28)	0.09 (0.17)	1.54 (0.29)
	Newly unemployed	191	3.65 (0.18)	3.62 (0.19)	6.30 (0.39)	−0.03 (0.19)	2.68 (0.33)
	Retired after workplace closure	252	3.32 (0.15)	3.09 (0.14)	4.81 (0.29)	−0.23 (0.16)	1.72 (0.29)
	Returned to workplace after closure	1 391	3.30 (0.07)	3.07 (0.07)	4.77 (0.12)	−0.23 (0.07)	1.70 (0.11)
	Returned to workplace after remote work	564	3.35 (0.10)	3.09 (0.10)	4.60 (0.17)	−0.26 (0.11)	1.51 (0.17)
	Switched to remote work	1 322	3.32 (0.07)	3.06 (0.07)	5.26 (0.12)	−0.26 (0.07)	2.19 (0.11)
	Remained in workplace	399	3.43 (0.13)	3.17 (0.12)	4.80 (0.23)	−0.25 (0.13)	1.63 (0.22)
	Remained remote	2 575	3.25 (0.05)	3.18 (0.05)	4.73 (0.08)	−0.07 (0.05)	1.55 (0.08)
	Workplace closed	192	3.50 (0.19)	3.31 (0.18)	5.86 (0.32)	−0.19 (0.21)	2.56 (0.31)
	Other	92	3.87 (0.29)	3.31 (0.26)	4.72 (0.42)	−0.56 (0.30)	1.40 (0.41)
Change from FUP1 to Autumn 2020	Remained retired	9 313	3.29 (0.03)	3.31 (0.03)	4.82 (0.04)	0.02 (0.03)	1.51 (0.04)
	Newly retired	369	3.12 (0.13)	3.15 (0.12)	4.55 (0.22)	0.03 (0.14)	1.40 (0.22)
	Newly employed	418	3.59 (0.12)	3.65 (0.13)	5.21 (0.24)	0.06 (0.14)	1.56 (0.23)
	Newly unemployed	187	3.60 (0.18)	3.58 (0.19)	6.26 (0.39)	−0.02 (0.20)	2.68 (0.33)
	Switched to remote work	3 824	3.27 (0.04)	3.14 (0.04)	4.91 (0.07)	−0.13 (0.04)	1.78 (0.06)
	Remained in workplace	354	3.55 (0.13)	3.23 (0.14)	5.01 (0.23)	−0.32 (0.14)	1.78 (0.22)
	Increased hours	306	3.33 (0.14)	3.18 (0.14)	4.61 (0.24)	−0.15 (0.16)	1.43 (0.24)
	Reduced hours	1 550	3.29 (0.06)	3.06 (0.06)	4.70 (0.11)	−0.22 (0.07)	1.64 (0.11)
	Workplace closed	178	3.45 (0.20)	3.21 (0.19)	5.88 (0.34)	−0.23 (0.21)	2.67 (0.33)
	Other	220	3.59 (0.17)	3.01 (0.16)	4.46 (0.26)	−0.57 (0.19)	1.45 (0.26)
Primary work location in Autumn 2020	Working from home	1 242	3.26 (0.07)	3.00 (0.07)	5.17 (0.12)	−0.26 (0.07)	2.17 (0.11)
	Working in non-home-based setting	2 319	3.35 (0.05)	3.10 (0.05)	4.71 (0.09)	−0.25 (0.06)	1.62 (0.09)
	Other location	263	3.49 (0.15)	3.17 (0.14)	5.35 (0.28)	−0.32 (0.18)	2.18 (0.27)
	Skipped	12 891	3.29 (0.02)	3.30 (0.02)	4.84 (0.04)	0.00 (0.02)	1.54 (0.04)
	Missing	4	3.50 (1.19)	4.75 (1.49)	5.25 (1.31)	1.25 (1.60)	0.50 (0.96)
Essential worker status in Spring 2020	No	2 389	3.29 (0.05)	3.09 (0.05)	5.15 (0.09)	−0.19 (0.05)	2.06 (0.08)
	Yes	1 783	3.41 (0.06)	3.13 (0.06)	4.69 (0.10)	−0.28 (0.06)	1.56 (0.10)
	Skipped	12 321	3.29 (0.02)	3.29 (0.02)	4.81 (0.04)	0.00 (0.02)	1.52 (0.04)
	Missing	226	3.40 (0.16)	3.33 (0.16)	5.54 (0.29)	−0.07 (0.19)	2.21 (0.28)
Work disrupted in Spring 2020	No	965	3.44 (0.08)	3.13 (0.08)	4.36 (0.14)	−0.30 (0.09)	1.23 (0.14)
	Yes	3 415	3.32 (0.04)	3.12 (0.04)	5.15 (0.08)	−0.20 (0.04)	2.04 (0.07)
	Skipped	12 321	3.29 (0.02)	3.29 (0.02)	4.81 (0.04)	0.00 (0.02)	1.52 (0.04)
	Missing	18	2.39 (0.31)	3.06 (0.51)	5.67 (1.02)	0.67 (0.58)	2.61 (0.88)

**Abbreviation: **CESD-10, Center for Epidemiological Studies Short Depression Scale. 

**Note:** Higher CESD-10 scores indicate more severe depressive symptoms. Individuals were classified as having depression when CESD-10 score was greater than or equal to 10. 

Table 4 presents adjusted ORs and 95% CIs for incident depression in Autumn 2020 by employment status. When examining changes in employment from Spring 2020 to Autumn 2020, newly unemployed individuals had over double the odds of depression (OR=2.22; 95% CI: 1.51–3.28) compared to those who remained retired. Additionally, those who switched to remote work had a 24% increased risk of depression (OR=1.24; 95% CI: 1.01–1.53), and those whose workplaces closed had 95% increased risk of depression in Autumn 2020 (OR=1.95; 95% CI: 1.32–2.88). Consistent patterns were observed for employment changes between FUP1 and Autumn 2020. We also observed that individuals working in non-home-based settings in Autumn 2020 had 21% decreased odds of depression compared to those working remotely (OR=0.79; 95% CI: 0.63–0.98). Similarly, essential workers had 25% reduced odds of depression compared to non-essential workers (OR=0.75; 95% CI: 0.62–0.91). Furthermore, those whose employment was disrupted in Spring 2020 had 65% higher odds of depression compared to those whose employment was unaffected (OR=1.65; 95% CI: 1.28–2.12). Overall, there were only slight differences between sexes, where women had greater risks of depression due to unemployment and workplace closure, and men had greater risks associated with work disruptions in Spring 2020.

**Table 4:** Odds ratios and 95% confidence intervals for incident depression in Autumn 2020 according to various employment factors in individuals without depression at Baseline or Follow-up one (sample size = 16 719; incident cases in Autumn 2020 = 2211)

Employment situation		No. cases	Model 1 OR (95% CI) – Overall	Model 2 OR (95% CI)		
				Overall	Females	Males
Change from Spring 2020 to Autumn 2020	Remained retired	1 182	1.0	1.0	1.0	1.0
	Newly retired	29	0.86 (0.58–1.28)	0.92 (0.60–1.42)	0.94 (0.52–1.68)	0.90 (0.47–1.70)
	Newly employed	50	1.30 (0.95–1.79)	1.40 (0.97–2.03)	1.62 (1.04–2.52)	1.02 (0.50–2.06)
	Newly unemployed	47	2.18 (1.54–3.09)	2.22 (1.51–3.28)	2.69 (1.60–4.52)	1.79 (0.98–3.24)
	Retired after workplace closure	36	1.16 (0.81–1.67)	1.25 (0.85–1.83)	1.78 (1.10–2.88)	0.73 (0.36–1.46)
	Returned to workplace after closure	180	0.92 (0.76–1.12)	0.91 (0.73–1.13)	0.89 (0.67–1.19)	0.95 (0.68–1.33)
	Returned to workplace after remote work	64	0.78 (0.59–1.05)	0.81 (0.59–1.10)	0.94 (0.63–1.40)	0.68 (0.41–1.13)
	Switched to remote work	206	1.18 (0.97–1.42)	1.24 (1.01–1.53)	1.18 (0.88–1.57)	1.32 (0.97–1.79)
	Remained in workplace	52	0.97 (0.71–1.33)	0.96 (0.67–1.37)	1.10 (0.67–1.79)	0.81 (0.47–1.38)
	Remained remote	314	0.94 (0.81–1.09)	0.99 (0.84–1.16)	1.06 (0.86–1.32)	0.88 (0.69–1.13)
	Workplace closed	39	1.75 (1.21–2.53)	1.95 (1.32–2.88)	2.19 (1.20–4.00)	1.79 (1.06–3.00)
	Other	12	1.05 (0.57–1.94)	1.20 (0.61–2.37)	0.53 (0.12–2.32)	1.56 (0.72–3.39)
Change from FUP1 to Autumn 2020	Remained retired	1 199	1.0	1.0	1.0	1.0
	Newly retired	47	1.00 (0.72–1.37)	1.05 (0.74–1.48)	1.20 (0.77–1.88)	0.87 (0.51–1.50)
	Newly employed	66	1.17 (0.88–1.54)	1.22 (0.88–1.68)	1.44 (0.97–2.13)	0.88 (0.48–1.59)
	Newly unemployed	46	2.14 (1.51–3.03)	2.19 (1.49–3.23)	2.67 (1.59–4.50)	1.73 (0.95–3.14)
	Switched to remote work	512	1.01 (0.88–1.15)	1.06 (0.91–1.23)	1.09 (0.89–1.32)	1.02 (0.81–1.27)
	Remained in workplace	49	0.98 (0.71–1.35)	0.92 (0.64–1.33)	1.11 (0.71–1.72)	0.67 (0.34–1.30)
	Increased hours	39	0.85 (0.60–1.21)	0.85 (0.57–1.26)	0.87 (0.55–1.37)	0.83 (0.36–1.95)
	Reduced hours	191	0.86 (0.70–1.04)	0.86 (0.69–1.07)	0.90 (0.66–1.21)	0.83 (0.60–1.14)
	Workplace closed	37	1.78 (1.22–2.60)	1.96 (1.32–2.92)	2.09 (1.11–3.93)	1.87 (1.11–3.15)
	Other	25	0.88 (0.57–1.34)	0.92 (0.57–1.49)	0.60 (0.27–1.32)	1.24 (0.68–2.27)
Primary work location in Autumn 2020	Working from home	180	1.0	1.0	1.0	1.0
	Working in non-home-based setting	294	0.83 (0.68–1.02)	0.79 (0.63–0.98)	0.83 (0.61–1.12)	0.76 (0.55–1.04)
	Other location	46	1.34 (0.94–1.92)	1.28 (0.87–1.90)	1.19 (0.65–2.17)	1.35 (0.80–2.27)
	Skipped	1 691	0.98 (0.81–1.18)	0.96 (0.79–1.17)	1.05 (0.79–1.38)	0.87 (0.65–1.16)
Essential worker status in Spring 2020	No	368	1.0	1.0	1.0	1.0
	Yes	219	0.75 (0.63–0.90)	0.75 (0.62–0.91)	0.77 (0.59–1.00)	0.72 (0.54–0.97)
	Skipped	1 585	0.85 (0.74–0.98)	0.84 (0.72–0.98)	0.84 (0.68–1.03)	0.84 (0.67–1.06)
Work disrupted in Spring 2020	No	98	1.0	1.0	1.0	1.0
	Yes	525	1.60 (1.27–2.02)	1.65 (1.28–2.12)	1.41 (1.01–1.97)	2.01 (1.36–2.98)
	Skipped	1 585	1.37 (1.09–1.73)	1.40 (1.09–1.81)	1.25 (0.89–1.74)	1.60 (1.08–2.38)

**Abbreviations: **CI, confidence interval; FUP1, Follow-up one; OR, odds ratio. 

**Notes:** Model 1 was adjusted for age and sex. Model 2 was adjusted for age, sex, highest attained education, marital status, household income, mortgage status, dwelling location, smoking status in Spring 2020, frequency of alcohol consumption since 1 March 2020 and chronic condition status. 

The number of cases is based on Model 1. 

Individuals were classified as having depression when CESD-10 score was greater than or equal to 10. 

The remission analyses among those who were depressed at Baseline or FUP1 but not in Autumn 2020 found that individuals who were newly employed between either survey wave had almost double the odds of remission compared to those who remained retired (OR=1.99; 95% CI: 1.57–2.53) (data not shown). Individuals who returned to their workplace in Autumn 2020 after working remotely or having their workplace closed in Spring 2020 had 24% and 15% reduced odds of remission in Autumn 2020, respectively, compared to those who remained retired. There was no difference in the odds of remission among those who worked in a non-home-based setting in Autumn 2020 compared to those who worked from home (OR=0.95; 95% CI: 0.79–1.14), among essential workers compared to non-essential workers (OR=0.95; 95% CI: 0.81–1.12), or among those who had their employment disrupted in Spring 2020 compared to those who were undisrupted (OR=1.04; 95% CI: 0.86–1.26).

## Discussion

Our longitudinal analyses of CLSA participants found that incident depression during the COVID-19 pandemic varied across several sociodemographic and employment characteristics. Across nearly all subgroups, depressive symptoms improved modestly from Baseline to Follow-up one but worsened from Follow-up one to Autumn 2020. Those aged 50 to 59 years, women, and those with chronic conditions or lower income experienced the largest increases in CESD-10 scores. Notably, those who were newly unemployed in Autumn 2020 had more than double the odds of depression compared with those who remained retired. In contrast, individuals who worked in non-home-based settings or reported being essential workers had reduced odds of depression. 

Employment-related determinants of depression appeared stronger among women. Women who became newly unemployed, experienced workplace closure, or transitioned to remote work had higher odds of depression. In contrast, these associations were smaller or not statistically significant among men. These findings underscore the heightened vulnerability of women to employment-related stressors during the pandemic, likely reflecting the dual burden of job disruption and gendered caregiving responsibilities. Other epidemiological studies have found that employment characteristics, such as working remotely, were more strongly related to adverse mental health outcomes in women than men during the pandemic.^30^ Additionally, several studies have reported increased psychological stress and adverse mental health outcomes among women compared to men, particularly those with additional caretaking or homeschooling responsibilities during the COVID-19 lockdowns.^31^,^32^

A compelling finding from our analyses was that mean changes in depression scores worsened across all sociodemographic characteristics, including age, marital status, highest education, household income, and mortgage status. Other epidemiological studies have similarly observed mental health declines.^33^,^34^ One exception has been the study by Wester et al.,^23^ who, in a sample of 36  478 UK participants aged 50 and older, reported decreased prevalence of sadness or depression during the pandemic. However, they observed a concurrent rise in loneliness, particularly among women, which may indicate different manifestations of psychological distress across settings. 

Interestingly, we observed reduced odds of depression among essential workers compared with non-essential workers. These findings contrast with reports of increased rates of burnout and stress among medical professionals and other frontline health care workers,^17^-^19^ and other essential lower-income workers who experienced inadequate COVID-19 safeguards and a lack of worker health protection.^35^ Given the demographics in the CLSA, which is comprised of those who would have been at least 50 years of age when the pandemic began, and who are predominantly Caucasian and more affluent, our analyses would underrepresent these types of essential workers. Furthermore, essential worker status was assessed early in the pandemic (Spring 2020) when essential workers may not have yet reached burnout. 

Similarly, those working in person in Autumn 2020 were less likely to be depressed. This may reflect that those who chose to continue working in person, assuming they had the discretion to make this choice, had fewer concerns about the health impacts of COVID-19. It may also reflect the mental health benefits of maintaining social connections^36^ and a lower chance of disruption to routine.^37^ Robust evidence exists that maintaining social connections throughout the pandemic protected against depression.^38^ However, it is important to note that remote work is not inherently detrimental to mental health. Its effects likely depend on the supports available to workers, such as opportunities for social connection, maintaining structured routines, and organizational guidance. Policy and workplace interventions that strengthen these supports may help to protect the mental well-being of remote workers, allowing them to benefit from the flexibility of remote arrangements without compromising psychological health. Our analysis categorized participants as working primarily at home, in the workplace, or “other,” the last of which may reflect hybrid situations but cannot be definitively interpreted as such. This inability to isolate the effects of hybrid work is a limitation, as hybrid arrangements may confer different mental health benefits than either fully remote or fully in-person work arrangements. In fact, previous work has shown that those who can work in hybrid situations have optimal trajectories for mental health compared to those who work fully remotely or fully in person.^9^ Furthermore, maintaining a regular schedule is important for one’s well-being, and those who have experienced disruptions in their work schedule or were required to shift to remote work may be at a higher risk for depression,^39^ a pattern that was evident in our study, where both workplace disruptions and transitions to remote work were associated with increased odds of depression. 

The employment-depression relationship is likely bidirectional. Depression can hinder job acquisition and retention,^40^ while employment changes can impact mental health.^41^ During the pandemic, social assistance aided those unable to work due to health issues, potentially introducing selection bias, excluding individuals with or at risk for severe depression from the sample. A further limitation concerns reverse causality and self-selection in work location during Autumn 2020. Individuals who opted to work in person may have differed systematically from those working remotely, for example, by having lower levels of anxiety, fewer health concerns, or stronger coping mechanisms. These underlying differences may have influenced both work location and the risk of depression, limiting the causal interpretation of our findings. 

It is worth noting that, due to the focus of the CLSA on midlife and older Canadians, this study excludes individuals younger than 50 years. Individuals below this age threshold also experienced stressors, including balancing childcare and homeschooling while adapting to remote work. Furthermore, essential workers with children faced additional difficulty finding childcare arrangements following school closures. The overwhelming stress experienced by younger workers is likely to have impacted their mental health and well-being. However, further research is necessary to understand better the impact of employment factors on depression within that population.

***Strengths and limitations***

We acknowledge that this study has limitations. The CLSA cohort is more affluent and less diverse than the general Canadian population, and excludes residents of the Territories and of First Nations reserves, individuals in institutionalized care and those who could not speak French or English. These criteria likely resulted in the recruited sample being healthier than the general population. Another limitation was the inability to adjust for physical activity levels during the pandemic. Although these data were collected during the Follow-up one survey wave, they were several years old at the onset of the pandemic and were based on the Physical Activity Scale for the Elderly,^42^ which provided only a limited snapshot of weekly activity. In addition, physical activity levels changed markedly during COVID-19 lockdowns and restrictions. As a result, pre-pandemic measures were not suitable for capturing these dynamic changes and were omitted from our analyses, which may have introduced residual confounding. 

In addition, although we defined employment transitions across survey waves, some heterogeneity within categories may remain. For example, differences in job quality or temporary disruptions were not fully captured by our measures. Additionally, these employment transitions cannot account for heterogeneity across occupations, as returning to in-person work may have carried different implications for health care workers compared to those in retail or other sectors. Furthermore, the classification of “essential worker” in our study was broad and did not distinguish between occupational groups such as health care, retail, or transportation, each of which may involve different levels of exposure risk, stress, and job security. Although standardized occupational codes are available in the CLSA Follow-up one data, they are part of the controlled access files that we were unable to obtain. Moreover, these codes were not collected in the COVID-19 surveys and could not be used to refine essential worker status during the pandemic. As a result, our estimates may obscure nuances within this category and could be influenced by unmeasured occupational characteristics. 

Another limitation was the relatively large loss to follow-up from the Follow-up one wave of data collection to the COVID-19 surveys, as nearly 20 000 participants from Follow-up one did not participate in the urgent CLSA COVID-19 questionnaires. Those lost to follow-up had slightly higher CESD-10 scores at Baseline and Follow-up one and were more likely to have lower household income and lower educational attainment compared with the analytical cohort (data not shown). To facilitate direct comparisons with Table 2, both analyses were restricted to participants without depression at Baseline or Follow-up one, thereby focusing on the same at-risk cohort for incident depression in Autumn 2020. This restriction ensured consistency across analyses but does not capture differences among participants with pre-existing depression, who may have been particularly vulnerable to dropout. Although the observed differences between the analytical cohort and the lost-to-follow-up cohort were modest, selective attrition of individuals with poor mental health and socioeconomic disadvantage may have led to an underestimation of the association between employment status and depression in our study. 

Our study also has notable strengths, such as the use of substantial longitudinal data obtained, which provides comprehensive information on sociodemographic factors and mental health measures, before and twice during the first year of the COVID-19 pandemic. This provided the opportunity to evaluate changes in mental health within the same individuals before and during the pandemic, thereby enriching the depth and breadth of our research. Unlike many cross-sectional studies, which are prone to temporal biases, our approach is more robust for studying the pandemic’s impact, particularly on depression. 

## Conclusion

In this large cohort of older Canadians, we observed worsening depression scores early in the pandemic and identified several employment-related risk factors for incident depression in Autumn 2020. Transitions such as becoming newly unemployed or experiencing workplace closure were associated with nearly double the odds of depression, whereas working in person and essential worker status were linked to lower odds. These findings highlight the need for public health and workplace policies that mitigate the impact of employment disruptions. Remote work arrangements should not be discouraged; rather, they should be supported through policies that promote social connection, stability and routine, helping to protect mental well-being while preserving the flexibility remote work provides. 

## Acknowledgements

This research was made possible using the data/biospecimens collected by the CLSA. Funding for the CLSA is provided by the Government of Canada through the Canadian Institutes of Health Research (CIHR) under grant reference: LSA 94473 and the Canada Foundation for Innovation, as well as the following provinces, Newfoundland, Nova Scotia, Quebec, Ontario, Manitoba, Alberta and British Columbia. This research has been conducted using the CLSA Baseline Comprehensive Dataset version 6.0, Baseline Tracking Dataset version 3.7, Follow-up 1 Comprehensive Dataset version 3.0 and Follow-up 1 Tracking Dataset version 2.2, COVID-19 Questionnaire Study Dataset version 1.0 under Application ID 2104024. The CLSA is led by Drs. Parminder Raina, Christina Wolfson and Susan Kirkland. Funding for support of the CLSA COVID-19 questionnaire-based study is provided by the Juravinski Research Institute, the Faculty of Health Sciences at McMaster University, the Provost Fund from McMaster University, the McMaster Institute for Research on Aging, the Public Health Agency of Canada/CIHR grant reference CMO 174125 and the government of Nova Scotia.

## Conflicts of interest

Margaret de Groh is the journal’s former Associate Editor-in-Chief and Paul Villeneuve is a former Associate Scientific Editor, but both have recused themselves from the review process for this article. The authors declare that they have no competing interests.

The study sponsors did not play a role in the study design, the collection, analysis, and interpretation of data, or the writing of the report. The CLSA team has approved the submission of this paper for publication.

## Authors’ contributions and statement

BF: Formal analysis, investigation, methodology, validation, writing—original draft, writing—review and editing.

YJ: Investigation, project administration, validation, writing—review and editing.

MdG: Conceptualization, funding acquisition, investigation, project administration, validation, writing—review and editing.

EFT: Conceptualization, funding acquisition, investigation, project administration, writing—review and editing.

IC: Investigation, writing—review and editing.

PJV: Conceptualization, investigation, project administration, methodology, supervision, validation, writing—review and editing.

The content and views expressed in this article are those of the authors and do not necessarily reflect those of the Canadian Longitudinal Study on Aging or the Government of Canada.

## Funding

Esme Fuller-Thomson gratefully acknowledges the support of the Canadian Institutes of Health Research (CIHR) grant #172862 (PI Esme Fuller-Thomson) and the Canadian Frailty Network. Brianna Frangione received funding from the Public Health Agency of Canada FSWEP program to support this research activity. 
